# Free will and the desire for suicide in mental illness

**DOI:** 10.3389/fpsyt.2022.909970

**Published:** 2022-07-15

**Authors:** Tobias Zürcher

**Affiliations:** ^1^Institute for Biomedical Ethics and History of Medicine (IBME), University of Zurich, Zurich, Switzerland; ^2^Gymnasium/FMS Thun, Thun, Switzerland

**Keywords:** assisted suicide, free will, decision-making competence, self-expression, autonomy, compatibilism

## Abstract

The desire to die brings about the most radical consequences that can occur in a human life. It therefore requires a high degree of justification. Questions have been raised as to whether this justification can be given in the case of a suicide desire in mental illness. Landmark court decisions and the practice of assisted suicide organizations make the justification of a mentally ill person’s suicide desire dependent on the desire not being an expression of the illness. This view is explained in detail and finally rejected as misleading. That argument is based on a conceptual analysis of the self, the nature of reasons for action, and the meaning of necessity with respect to personal autonomy. Against this background, it is shown that it is irrelevant for the assessment of the desire to die whether it has been causally brought about by the mental illness. On the other hand, what matters is whether the person has an internal reason that gives importance to his or her desire. This is to be distinguished from external, normative expectations of a person’s “normal” desires. An internal reason that justifies the person’s concern must give expression to who the person essentially is and what the person fundamentally cares about. Three objections to this view are formulated, critically evaluated, and rejected. From these considerations it follows that a professional assessment of the desire to die of mentally ill persons must consist primarily in clarifying whether the desire to die fulfills the stated conditions for freedom, irrespective of the mental illness.

## Introduction

Should a person suffering from mental illness be entitled to request assisted suicide? Does such a decision have any moral merit? The answer to this question depends on an assessment of various assumptions that lie at different levels of generalization. From a high-level perspective, it matters whether we think assisted suicide is morally justified at all. Even one level higher, the question would be whether suicide is morally justified. For the purpose of the argument of this paper I shall assume that there are justifiable cases of suicide and suicide assistance.

A classic argument for the justification of assisted suicide is expressed by Dworkin et al. in their *Philosophers Brief* presented to the United States Supreme Court. The authors claim that “every competent person has the right to make momentous personal decisions which invoke fundamental religious or philosophical convictions about life’s value for himself” (2) and this should include the decision about how we want to die, as a highly personal, almost sacred matter. To not respect this will would be particularly cruel. One way in which this will is disregarded is by not giving help in implementing this decision to those who cannot put it into practice on their own – or, depending on one’s point of view, do not want to make it without help.

If we presuppose this position – as I will do in what follows – the question arises at an intermediate level as to who can (morally) claim assisted suicide and in what circumstances. In this regard, the position of Dworkin et al. is instructive in another respect, in which the authors outline the conditions for claiming this right: “people may make such momentous decisions impulsively or out of emotional depression, when their act does not reflect their enduring convictions; and it therefore allows that in some circumstances a state has the constitutional power to override that right in order to protect citizens from mistaken but irrevocable acts of self-destruction.” (2) The decision to die is morally respectable when it is an expression of urgent and fundamental, highly personal desires. However, a mental illness such as major depression could now be one of the reasons why the person is not competent to make this decision. What is true of depression could be true of many other mental illnesses, insofar as they similarly imply that the desire to die is not grounded on an “enduring conviction.” (2) We can thus draw from this to formulate an objection to assisted suicide for people with mental illness based on a categorical difference between mental and physical illness (or health):

Even if assisted suicide may be justified under ordinary circumstances, the case is different in the presence of mental illness. The desire to die and the mental illness are usually so closely related, possibly causally, that the autonomy of the decision to die is at least very doubtful. It therefore follows from a precautionary principle to treat the desire to die of a mentally ill person categorically differently from the desire of a healthy (or somatically) ill person.

This hypothesis implies an assumption which is often not explicitly stated, but which must be examined in more detail later: A competent decision that deserves moral respect, e.g., a decision that involves choosing or refusing therapy, justifies behavior insofar as it is done out of free will only. We are thus at a third level of generalization when we ask whether it is possible for a person with mental illness to make a free and (in all other essential respects) competent decision to die. This is the level of specification this paper is concerned with. I will elaborate on the above-mentioned hypothesis in see section “A competent and free decision to die when mentally ill?” and then argue in see section “Discussion” that the hypothesis is important empirically but misleading from a normative point of view. Building on the work on the notion of the self and normative reasons for action, I propose that from a moral point of view, it is not relevant whether the desire to die is caused by a mental illness.^1^

## A competent and free decision to die when mentally ill?

A decision for or against medical treatment requires moral justification. The doctrine of informed consent demands that a competent patient be sufficiently informed to reach a voluntary decision (3). A person is decision-making-competent (DMC) if he or she understands the relevant information (especially about his or her condition and the treatment options under discussion with their respective benefits and risks), appreciates the relevance to his or her situation, is able to rationally weigh the options, and can finally communicate his or her decision (4). A justified decision is one that is truly autonomous and reflective of its underlying values. I have argued elsewhere that free will is also a condition of a justified decision and that DMC, in particular an appropriate interpretation of the aspect of “appreciation,” includes the condition of free will (5). The appreciation condition is only met if the person can sufficiently identify with his or her own desire and thus approves of the desire free of internal or external constraints. In this respect, the conditions of DMC and free will (as it will be established in see section “Does necessity threaten freedom?”) do overlap.

Can these conditions be met in the case of a mentally ill patient’s desire to die? Let us first consider a decision of the highest Swiss court (*Bundesgericht*) (6). The court had to decide whether a 53-year-old man suffering from severe bipolar affective disorder could be given the prescription drug sodium pentobarbital (NaP) as part of an assisted suicide with the assisted suicide organization *Dignitas*. The man had already committed two suicide attempts and was repeatedly treated as an inpatient. The court ruled that the prescription requirement for NaP must be upheld, and the man could not be given the drug because a private organization could not check as well as a licensed psychiatrist whether the drug was indicated. Two parts in this ruling are particularly interesting, when the court states:

1. “It is important to recognize that an incurable, permanent, severe mental impairment, similar to a somatic one, can cause suffering that would make the patient’s life seem no longer worth living in the long term.” And,
2. “However, utmost caution is required in this respect: A distinction must be made between the desire to die, which is the expression of a treatable mental disorder and calls for treatment, and the desire to die, which is based on a self-determined, well-considered and lasting decision by a competent person (“balance suicide”), which must be respected where appropriate.” [(6), my own translation].

In a similar vein, the German Federal Constitutional Court (Bundesverfassungsgericht) ruled (7) that the decision to commit suicide was a fundamental expression of the right to self-determination. However, such a decision must arise independently of a mental illness and be exercised independently of it. This would be not the case if the decision was induced by an acute mental illness or if the desire was not of a certain permanence and firmness. (7) In light of such court decisions, even Switzerland’s largest assisted suicide organization, Exit, has provided assisted suicide for patients with mental illness only in very rare cases. Exit states that assisted suicide can only be considered if it is a “permanent desire to die” that is “well-informed, well thought-out and not, for example, the result of a momentary depressive mood or crisis” (8).

What these positions (considered exemplarily) have in common is that the justification of a decision is made dependent (among other criteria) on the permanence and independence of mental illness. At the core is the following idea: the desire must not be an “expression” of the illness, which would be incompatible with the decision being made competent and autonomous.

But what does that mean exactly? We can imagine a whole spectrum of interpretation here. On the one hand, there is the idea that any connection between mental illness and the desire to die under any possible circumstances already excludes freedom and decisional competence, and that even the expression of the desire is an indication that the person is incompetent (9). This overall pathologizing of the desire to commit suicide is contrasted, on the other hand, with the idea that a person can decide competently and freely at any time (even, for example, in a deeply depressed mood). The position presented above stands somewhere in between. It assumes a certain kind of ideal of an objective observer of the self upon itself. It assumes that a competent and free decision is possible only if this impartial thinker, uninfluenced by their illness, develops, weighs, and finally approves or rejects his desire to die. This is what it means to say that the decision is not an expression of the disease, but independent of it. This position conceptually allows the case of a mentally ill and competent person but sets a very high bar this to be actually possible. Is this convincing? Does a decision that is an expression of mental illness preclude a competent and free decision?

## Discussion

### Mental illness, suicidal ideation and suicide

Suicidal ideation, suicide, and mental illness are empirically related. It is reasonable to assume that not every suicide is an expression of mental illness (10). To claim the opposite would mean to exclude the possibilities of non-pathological suicidal thoughts by definition; such a conceptual framework would mean to limit decisively the spectrum of autonomous decision of rational beings. Nevertheless, there is strong evidence that mental illness is strongly correlated with suicidal ideation, that is, all sorts of thoughts and contemplations, desires, with death and suicide, suicide attempts, and actual suicides (11). This correlation is particularly strong for depression and anxiety disorders (12).

These correlations alone do not tell us that mental illness actually diminishes or eliminates autonomy; however, it does provide us with initial evidence that this may be the case (13). The impediment can potentially be identified in all aspects of decision-making competence. It is conceivable that a cognitive impairment caused by a mental illness makes it difficult or impossible for a patient to understand the relevant information for a therapeutic decision or for the decision for or against suicide in the first place. In a similar way, it is possible that this could impair the ability to reason or to relate the facts to one’s own life (appreciation). The existence of these cases is not to be disputed in any way in what follows.

Beyond these constellations, however, the question remains whether a decision to commit suicide is not competent and free if the mental illness makes it seem necessary or at least seems to strongly support it. The goal of this paper is to argue that acting on desires to die can be autonomous despite not being casually independent from the influence of mental illness. The courts’ reasoning and the hypothesis formulated above (see section “Introduction”), however, are rather ambiguous. While they could be interpreted as not contradicting the (aimed) conclusion just stated, they hold considerable potential for misunderstanding based on which a person’s autonomy is rashly denied. The danger, as I see it, is that finding a robust causal relationship between the desire to die and mental illness, is interpreted as a clear sign of the person’s lack of autonomy. Therefore, in addition to the *descriptive* finding of evidence for the causal relationship between the illness and the desire to commit suicide, the *normative* question remains: Could a desire to die that is produced or at least strengthened by the mental illness be free and competent?

### Does necessity threaten freedom?

In order to examine this normative question about the connection between illness and the desire for suicide, we must try to clarify the relationship between the necessity of a decision and its voluntariness. In other words: Can anything (an event, a desire, a decision, etc.) appear inevitable or even be necessary (in the sense of “being an expression of X”) and at the same time be free?

This leads back to the classical philosophical debate about freedom of will. Here the idea that necessity excludes free will can be illustrated by a short thought experiment and a subsequent argument. The physicist and philosopher Pierre-Simon Laplace illustrated the idea of the universal existence of necessity (i.e., determinism) with this thought experiment:

“We may regard the present state of the universe as the effect of its past and the cause of its future. An intellect which at a certain moment would know all forces that set nature in motion, and all positions of all items of which nature is composed, if this intellect were also vast enough to submit these data to analysis, it would embrace in a single formula the movements of the greatest bodies of the universe and those of the tiniest atom; for such an intellect nothing would be uncertain and the future just like the past would be present before its eyes.” [(14), p. 4].

The thesis of universal determinism is a scientific one and not yet a thesis about free will. However, starting from the idea of determinism we can establish a connection to freedom by considering Peter Van Inwagen’s *consequence argument*:

“If determinism is true, then our acts are consequences of the laws of nature and events in the remote past. But it is not up to us what went on before we were born, and neither is it up to us what the laws of nature are. Therefore, the consequences of these things (including our present acts) are not up to us.” [(15), p. 16].

It is important to see that the consequence argument is formulated as a conditional. The *consequent*, namely the impossibility of free will, depends on the *antecedent*, the existence of determinism, being true. We don’t know whether determinism is true or false, this is a scientifically open question (16). However, we can leave this question unanswered because what concerns the relation of necessity and a particular desire is this: Would determinism (and therefore necessity) exclude freedom, if it existed? If it turns out that it does not, then *a fortiori* any weaker influence than necessity, such as mere reinforcement, or increase of the probability, do not have to rule out freedom. In relation to the connection between mental illness and the desire to commit suicide, this would mean that a decision to commit suicide can be free even if the illness reinforces, contributes to, or even necessarily causes the desire to do so.

Opposing this incompatibilistic understanding of necessity and freedom (or determinism and free will) stands the idea of compatibilism, which assumes that freedom is not threatened by determinism (17) or even presupposes it (18). The compatibilist understanding can be traced back to the Stoics, among others, and was defended by Thomas Hobbes or David Hume. Harry Frankfurt is one of the most important contemporary proponents of compatibilism. I have argued elsewhere that the compatibilist understanding is convincing for medical ethics, especially in the context of psychiatry and psychotherapy (19). If we were to follow incompatibilism (of determinism and free will), we would have to demand different conditions for an autonomous decision. It would be a concept of autonomy that presupposes the essential conditions of compatibilism and postulates additional conditions, such as that the autonomous decision must not be caused at all or (*ex nihilo*) caused by the person him- or herself. In the following, I will draw on key points of Frankfurt’s theory, without defending it against all possible objections. However, the examination of the arguments in the following two sections aims to show that a Frankfurtian approach is a convincing interpretation of a mentally ill person’s desire to die. The burden of proof for a more demanding incompatibilist conception of autonomy therefore lies with its proponents.

The Frankfurt account assumes that a person is autonomous if the motivation to act is in accordance with the person’s set of mental states. Different “coherentist” theories focus attention on different conditions that must be met for this coherence of desire and mental state to be achieved [cf. (20), 2]. These distinctions have no bearing on the issue at hand. However, we would reach a different conclusion (at least in some cases) if we were to require a stronger consideration of external reasons for personal autonomy. One famous such approach is advocated by Fisher and Ravizza (21). They argue that the autonomous person must be able to adequately assess (moral) reasons, including those of “external” origin (i.e., outside the person’s set of reasons). This standard requires that a person’s reasons must have a minimal relation to reality (as shared by an external observer). At this point, it is not possible to argue at length for the merits of the Frankfurt approach. The reason for my choice is essentially this: I share the view, as defended in see section “The Desire to Die as Expression of the True Self?”, that subjectively important reasons, which are to be respected morally, are internal reasons of a person or must upon appropriate reflection be able to become internal reasons. A persons autonomy does not depend on how it came to be that we are who we are, nor that our motives are rational by some external standard, nor that they represent the state of the world particularly well.

### The freedom of the self and its fundamental desires

Frankfurt sets out a quite simple definition of what it means to have free will. Whereas freedom of action consists in being able to do what we want to do, we enjoy free will when “when what we want is what we want to want.” [(17), p 15]. *Persons* are those beings who can sometimes enjoy this kind of freedom. When we manage to perform an action that we do for exactly those reasons that we want and approve of, we are as free as we can possibly be.

This view belongs to a group of “self-expression theories.” These theories assume that freedom is bound to essential characteristics of a person, which are being expressed in a free act of willing. A free desire expresses what the person is “at heart” or at least does not conflict with these fundamental aspects of the person. The concept of the self that underlies this position does so without any particular metaphysical presuppositions. No cartesian ego or unchangeable soul is assumed; the self simply consists in the “cluster of attitudes that specify what matters most to her and what is for her most worth pursuing.” [(22), p. 785]. These fundamental attitudes are the basis of the practical reasons for action. According to Frankfurt, what we care for is the ultimate source of normativity. A notable variant of caring is thereby love [(23), p. 11]. There are some similarities, but above all many differences in what people care about and what they love. This is because the conditions for care and love, the causal stories that lead to them, are extremely diverse. To be sure, there are biological-empirical reasons that lead to relative consistency in some aspects of human caring; Frankfurt mentions the love of one’s children or the love of being alive (p. 30). But even in these cases, the biological variance is conspicuous: not all people love children, not even their own, and not all are equally intensely attached to life or are willing to weigh the value of their own life against another value in different ways. For all our caring and loving, however, the following is true: We have only very limited or no control over what we care about or what we love. In most cases, it just happens to us. However, the necessity of our caring (for a person, a thing, or a project) does not diminish the power of our caring at all. A person’s freedom is grounded in the fact that he or she can reflectively refer to his or her desires against the background of what he or she cares about. A free person is not simply exposed or subjected to any desire he or she has but is able to approve those desires that express his or her values, which in turn are an expression of who the person is and what he or she cares about.

### A fundamental desire to die in mental illness: Three objections

Could a desire to die satisfy the requirement of reflectivity just described and do so even in the face of mental illness? I now discuss three objections to the possibility that a desire to die is *both* an expression of what the person is and cares about and brought about and shaped by the illness. In (1), closely related to the idea of necessity (see section “Does Necessity Threaten Freedom?” above), we raise the question, whether the causal origination of a desire to die by mental illness nullifies freedom. The second objection (2) concerns the assumption that the desire to stay alive must be attributed to the “deep self” and that mental illness, as it were, overshadows this desire and therefore in some sense devalues the desire to die. Finally, (3) we examine the significance of the changeability of the desire to die and the consequences that result from this for the merit of this desire.

#### In what way is causation relevant to the autonomy of a person’s desire?

Let us look more closely at the first objection, which we can formulate as follows: Many mental illnesses, especially major depressive disorders, have a significant impact on the way we judge our quality of life and the overall value of our own life, both in retrospect, with regard to the current state of affairs, and with regard to the future, including our plans and hopes (24). Consequently, our assessment is not realistic and does not correspond to our actual interests. The objection can be presented in many different narrative framings, such as a biologically inspired one according to which the disease results in such massive neuronal effects, e.g., on the functioning of neurotransmitters, that the emotional low, the hopelessness is a direct expression of it. A somewhat popularized version of this narrative reads like this: “Dopamine has taken over our souls” [(25), p. 198]. This could be the case, for example, if (substance) addiction has altered our pleasure-pain-threshold in such a way that we now (at least in the absence of using) fall into an anhedonic depressive state. The conclusion would be: a desire to die under these circumstances is not an expression of what the person is and cares about.

We must carefully distinguish between two aspects when assessing this objection: First, this is about the description of physical states and causal relations. Whether they exist is an empirical question and, insofar as they exist, there is no point in arguing against it. Second, the *conceptual* issue is whether these correlations (and causalities) are a normatively significant deviation from some sort of normal state and, in this respect, threaten freedom. In answering this question, we need to differentiate precisely: It may well be that, looking at the person and her fundamental evaluations, we conclude that the desire to die exists but is alien to him or her in a crucial way or incoherent with other fundamental desires. In this case, the disease would have a kind of dominant influence on the volitional process that defies the reflection of the person herself and makes it impossible for her to identify and weigh her important desires. This may be so; but whether this is the case does not depend on the fact that the desire to die is *causally* originated. We are all governed by causal processes. Our desires, be they mundane or those considered “pathological,” be they in some way beneficial to our health or self-destructive, they can all be described in the same biological or psychosocial categories. This becomes clear, among other things, when we realize how close a “neuronal story” of health and disease are to each other (26). This does not mean that they are all to be evaluated equally in terms of their capacity to make us happy or according to any moral criteria. But it does mean that we could not discount these desires by reference to their causal force or necessity. To do so would affect all our desires; so the objection misses the point. The causation of a desire does not destroy freedom; it only means that we are not all-powerful [(17), p. 16]. The mere fact that a desire can also be caused by an illness – be it mental or physical – does nothing to clarify the ethical question of whether the desire deserves to be respected. Autonomous desires can also be caused (as possibly every desire is); in fact, the causes of a desire are usually more noticeable to us if they are not of an ordinary nature.

#### The desire to die as expression of the true self?

Can the desire to die, if it is brought about by the mental illness, be an expression of the true self? Doesn’t the “true self” always want to stay alive? Frankfurt also gives thought to this: “Even of people who commit suicide because they are miserable, it is generally true that they love living. What they would really like, after all, would be to give up not their lives but their misery” [(23), p. 47]. The mentally ill patient could thus act under the pressure of the illness, but this pressure weighs on him or her as something alien, which is to be distinguished from the desire to stay alive as something of his or her own. (This assessment is analogous to the argument that social pressure, e.g., from relatives, could make a suicide decision inauthentic). These concerns can be framed in terms of the following argument: (P1) A desire to die is justified only if it is an expression of the fundamental values and attitudes of the person (i.e., of the deep self). (P2) The desire to die is caused by mental illness. (P3) A desire cannot be both being caused by mental illness and be an expression of the deep self. (C) Therefore, the desire to die of a mentally ill person is not justified.

We assume in the following that (P1) is true. (P2) is epistemically demanding. It involves the problem that a person may have multiple (independent) sufficient reasons for a particular desire (and action), which itself may have different (independent) causes. However, we also assume for the sake of argument that (P2) is – at least in most cases – true. However, we have good reasons to doubt the truth of (P3). The idea behind it follows Aristotle’s thoughts on the question of whether or not someone who throws parts of the ship’s cargo overboard in a sea storm to save the ship from sinking does so voluntarily. Aristotle thinks: “Such actions, then, are mixed, but are more like voluntary actions; for they are chosen at the time when they are done, and the end of an action is relative to the occasion.” [(27), p. 39, 1110a]. Whether the desire to die gives expression to what we fundamentally care about is also highly contextual. (This may complicate and confuse situations in frustrating ways, but it is simply the consequence of the diversity of possible contexts). At this point, it is important to see that the (Frankfurtian) understanding proposed here is open to which diagnostic methods to use to characterize a person’s desires. Compatible with this and promising for the question at hand could be, for example, a narrative approach (28), which, however, need not be in competition with DMC criteria, but helps in particular to clarify the aspect of “appreciation” by making it clear what someone cares about.

The search for the “true” reasons for action of the self leads back to the fundamental question of what it means to have a reason. When does A have a reason to do X? In this question the *internalists* (advocates of *reasons internalism*) compete with the *externalists* (29). The basic idea is this: Internalists claim that a reason for action must always be related to an individual’s motivation. Accordingly, R is a reason for A to do X if and only if R motivates A in some way to do X. Externalists deny this connection. This distinction is illuminating for our question without requiring us to definitely take a position. It serves to help us distinguish more clearly which *normative expectations* we must categorize as external to a person and which we can reconstruct as internal to the person himself or herself.

Finlay has convincingly argued that there is a connection between the importance of a reason for the subject and whether it is external or internal (30). Important reasons are those that exert normative force on us. These are internal reasons; an external reason (given it exists), on the other hand, is an unimportant reason. We ourselves decide what is important to us. Finlay holds: “Our intrinsic concerns are the source of importance for us not by constituting intrinsically important facts, but by making it the case that their objects matter intrinsically to us.” [(30), p. 17]. The reasons, which are moreover not only important but express who a person is and what is fundamentally important to him or her, can only be internal reasons – it would be downright bizarre to leave these innermost qualities and valuations, to external attribution. Moreover, the importance of a reason for the person lies in the fact that the person is holding this very reason and is not directly dependent on how things are (the idea of world peace motivates someone; another is afraid of imaginary ghosts). This subjective sovereignty over the importance of reasons, however, does not exclude that we can be mistaken about reasons or that we can fall prey to a paralyzing lack of imagination. Bernard Williams, one of the most prominent theorists of internalism, has therefore suggested that a person has an (internal) reason to do X if he could come to realize through sound deliberation that R is a reason to do X [(31), p. 110]. In this context, it is necessary to determine normatively what expectations we have of these deliberative abilities. (Does it concern the ability to avoid obvious errors and to recognize incoherencies in one’s own opinions or does it concern complex deductions from one’s own belief system? Our answer to this will depend on what demands we make on humans as *rational* beings).

A mentally ill person’s desire to die can, under certain circumstances, be a kind of error; an obvious case of this kind could be a delusion which, at a given moment, leads to a fundamentally wrong assessment of one’s own condition and therefore suggests irrational actions – even relative to one’s own fundamental desires. A deep, debilitating sadness that strikes a person in a surge could mean that the person is disregarding his or her own fundamental desires at that moment. As mentioned above, physiological findings can serve as signs that a person’s autonomy may be at risk. The more precise our understanding of these signs and their underlying mechanisms, the better we can weigh the evidence for interference with autonomy. A fascinating example of such an interplay between volition formation and autonomy of desire can be found in the phenomenon of fear learning. Recent research seeks to better understand the functional interplay between central and autonomic nervous systems (32). By better understanding the neural concomitants of fear conditioning, diagnostic protocols could be improved, and ultimately therapeutic approaches could be enhanced, as fear is ubiquitous in mental illness. Should we, as the above-mentioned study suggests, through measures of heart rate variability better succeed in clarifying the relationship between psychological and physiological processes, then we can also better evaluate the autonomy of the decision. One of the tasks of sensitive diagnostic or therapeutic work may be to find the sound-deliberative route to temporarily blanked-out desires and reasons for action. Knowing dominant forces that influence the formation of volition helps us in our search for a person’s internal reasons. This diagnostic task is therefore a support of an internal process. It is not a matter of convincing the person, but of making him or her aware of reasons that can develop a force – namely, precisely when they, now that they have been uncovered, as it were, emerge in the person’s set of internal reasons. Reasons can be offered “from outside,” but a person cannot be forced to internalize a reason. This applies equally to reasons that exert social pressure (exerted by relatives or as an expression of social expectations) for suicide as well as to any ethically motivated reasons against suicide. A reason that counts is an internal one or one that may become internal by incorporating it in the set of reasons that matter to a person. An external reason based on a statement along the lines of “everyone basically wants to stay alive” is at best a (empirically convincing) generalization or expression of a hope. But it is not something that directly represents the self until it has become internal. Therein lies part of the tragedy of a person’s desire to die, willing (as Aristotle meant it) to throw the load overboard. Therefore, there is the possibility of a rational desire to die, that is, a desire that may be consistent with the person’s fundamental beliefs and attitudes. The wish may be conditioned by circumstances that no one – at last the person herself – would have wished for. But this regret should not obscure the fact that it is nevertheless exactly what the person wants. Premise 3 of the argument presented above therefore proves to be poorly supported and the argument appears to be inconclusive.

#### Unchangeability as a sign of authenticity of a desire?

Finally, let us turn to the last objection, which states that a desire to die on the part of a mentally ill person is problematic because it arises from an unstable state and is therefore itself temporary. Thus, the fear is, as is also expressed in the court decisions, that the person who had committed suicide would later have wanted to get well and stay alive. This consideration is expressed, for example, by Mehlum et al. when they argue: “[W]ishes for death or suicide, even when clearly articulated by the patient to doctors or next-of-kin, and even if it represents the true will at that very moment, this desire or wish for death will likely change in many of these cases. […] such an articulated death wish, can be a symptom of the disorder and may in reality convey several other possible messages, that have more to do with the patient feeling abandoned, disappointed or angry. It may also convey a wish for help to live rather than a wish for help to die.” [(33), p. 6]. How do we assess this argument from changeability? First, it should be noted that the scope of the argument is limited. A disease may persist, a therapy may be unsuccessful, and the patient may die without having first recovered and given up his suicidal desire. But there is another problem with the connection between the importance of a desire and its changeability. If we start from the “healthy” example of a desire, for instance, to stay alive or to pursue a project, we see that this desire does not derive its moral weight from the fact that it is permanent or unchangeable. For it is very possible and empirically often the case (and to verify only in retrospect) that such a desire changes or is abandoned; people get sick, just as sick people can also get well and a person may change his or her mind or even change herself to some degree. The different weight we give to what is called “healthy” according to common sense and to what is called “ill” stems from the different valuation of illness and health (and the corresponding states). There is nothing wrong with this evaluation; the vast majority of human beings value health more than illness. However, it is important to see that we cannot deduce anything from this regarding the authenticity of a desire. We must be aware that this assessment of desires implies further evaluations. For example, the normative issue of what priority, role, or importance an illness has or should have in one’s life, or what effort, endeavor, or strain someone should take on in order to recover or to regain full health. But all this should be decided by the patient. If someone else would decide for him or her, this would mean applying a kind of paternalistic principle that obliges perseverance, declares certain life goals to be binding, and denies the affected individual his or her own weighing and sense-making. It is not impossible to argue in favor of this; but in this case it must be admitted that the balancing is done at the expense of autonomy.

Similarly, just as the authenticity of the desire is not necessarily dependent on its unchangeability, neither is it dependent on its unambiguity. Shaw et al. point out that the desire of a competent person can be ambivalent (13). In principle it seems rational to avoid those decisions which, in case of error, cause the greatest costs or which cannot be remedied. However, if we exclude for a moment the most ambivalent or unstable desires, it is clear that prudence has its price on both sides. For ultimately, the considerations of the stability and permanence of a desire again reveal what is at stake: do people who express the desire to die but do not *really* want to die receive the help to which they are entitled, and do we respect the free decision of mentally ill persons and provide them with help and assistance they need? It lies in the nature of decision-making in uncertainty that we cannot completely minimize both sorts of errors. Efforts to avoid one error easily lead to increasing the other. For obvious reasons, the “cost” of error is very high when a person has died who, as a matter of fact, would not have wanted to die. This justifies the application of a precautionary principle, as long as it does not result in the “cost” of the opposite error exceeding the “cost” avoided. The ideal to strive for is to minimize the “costs” of the accumulated errors due to the applied standard of due diligence. It is ultimately a therapeutic or diagnostic question to determine when a change of mind may constitute evidence of the person’s lack of autonomy. It is plausible to assume that at least a rapid change of mind constitutes *prima facie* evidence of the absence of autonomy, especially if the person cannot explain his or her reasons properly or if the wish seems alien to him or her in some way. We should make every effort to evaluate, as sensitive and careful as possible, the degrees of autonomy and the extent of assistance offered. This requires a high level of professional competence which must be grounded in conceptual and normative considerations of the self and its fundamental values.

## Conclusion

Mental illness can disrupt the decision-making process to such an extent that it is appropriate to characterize the decision as an expression of the illness – rather than of the person’s fundamental desires. However, if the person is able to take into account the weight of the illness (and its prospects), so to speak, in his or her own deliberation, then he or she can be autonomous. This is true even if the disease had a significant influence on the formation and weighting of the reasons. The insight that a desire to die can be authentic even if it is produced by the disease and is changeable is the result of conceptual analysis. As such, it can help to better understand the problem at hand and to critically question (implicit) normative assumptions. However, it does not make the work of medical professionals, whose job it is to assess the desires to die of mentally ill patients, any easier. Their role is to support the patient in finding a sound deliberative route to what may be non-present but fundamental desires. In this respect, mental illnesses, for empirical reasons, certainly require special caution. On the other hand, the “normative risks” must also be considered: The desires of the mentally ill must not be systematically “discounted,” for example, because normative expectations would translate into supposedly descriptive assessments of competence. We must not set the standard expectation that a person will wait for recovery and hold out until then if this conflicts with what the person fundamentally cares about. Nor must the moral duty to beneficence mean establishing health as a normative baseline state and qualifying desires formed in any other state as pathological.

It is not to deny that mental illness in many cases may lead to a desire that disguises who the person is and what he or she cares about. The task of diagnostics and therapy is then to disentangle this web of contingency as best as possible. As long as mental illness is stigmatized to a considerable extent, it will not become easier to determine the authenticity of the desire. A necessary conceptual connection between mental illness and the insincerity or inauthenticity of even a temporary desire to die, however, does not exist. Ignoring this would mean a simplification that can be cruel in precisely the sense that Dworkin et al. pointed out, even though they did not have the case of mental illness in mind.

## Author contributions

The author confirms being the sole contributor of this work and has approved it for publication.

## Conflict of interest

The author declares that the research was conducted in the absence of any commercial or financial relationships that could be construed as a potential conflict of interest. The reviewer FR declared a shared affiliation with the author TZ at the time of review.

## Publisher’s note

All claims expressed in this article are solely those of the authors and do not necessarily represent those of their affiliated organizations, or those of the publisher, the editors and the reviewers. Any product that may be evaluated in this article, or claim that may be made by its manufacturer, is not guaranteed or endorsed by the publisher.

## Footnotes

1 I will not consider an even more specific level that addresses who exactly should be involved in assisted suicide or whether there should be a legal right to assisted suicide.
